# Construction of a prognostic signature of autophagy-related lncRNAs in non-small-cell lung cancer

**DOI:** 10.1186/s12885-021-08654-2

**Published:** 2021-08-14

**Authors:** Xinyang Zhang, Yu Cao, Li Chen

**Affiliations:** 1 Department of Pathology Anatomy, Medical College of Nantong University, Nantong University, Nantong, 226001 Jiangsu China; 2 Third People’s Hospital of Nantong, Nantong, 226001 Jiangsu China

**Keywords:** Autophagy, Cancer, lncRNAs, Signature, Immune infiltration

## Abstract

**Background:**

Autophagy inhibits tumorigenesis by limiting inflammation. LncRNAs regulate gene expression at various levels as RNAs; thus, both autophagy and lncRNAs are closely related to the occurrence and development of tumours.

**Methods:**

A total of 232 autophagy-related genes were used to construct a coexpression network to extract autophagy-related lncRNAs. A prognostic signature was constructed by multivariate regression analysis. Kyoto Encyclopedia of Genes and Genomes (KEGG) pathway analysis was applied to analyse enrichment in cancer-related pathways. Immune infiltration analysis was used to analyse the relationship between the prognostic signature and the tumour microenvironment.

**Results:**

Nine autophagy-related lncRNAs were used to construct a prognostic model for non-small-cell lung cancer. The median risk score was used to discriminate the high- and low-risk groups, and the low-risk group was found to have better survival. Because KEGG pathway analysis showed that the prognostic signature was enriched in some immune pathways, further analysis of immune infiltration was conducted, and it was found that the prognostic signature did play a unique role in the immune microenvironment. Additionally, the prognostic signature was associated with clinical factors.

**Conclusion:**

We constructed a prognostic model of autophagy-related lncRNAs that can predict the prognosis of non-small-cell lung cancer.

## Introduction

Lung cancer is one of the most serious malignancies that threatens health, and its incidence has increased, mainly owing to the increase in smoking, a known risk factor [1]. Autophagy is a conserved catabolic cellular process in which damaged or unusable proteins or other cytoplasmic components are transported to double-membrane vesicles (autophagosomes) and then enter lysosomes or vacuoles for degradation [2]. The level of autophagy is a key threshold for promoting cell survival or inducing cell death in response to environmental stress [3–5]. Autophagy plays a related role in tumorigenesis and anticancer treatment by modulating inflammation, hypoxia, immunosuppression and metabolism in the tumour microenvironment. In particular, impaired autophagic flux is associated with chronic inflammation, immunosuppression, matrix formation, cancer stem cell generation, angiogenesis, metastasis, and metabolic reprogramming in the tumour microenvironment. The tumour microenvironment is composed of a variety of immune cells, mesenchymal cells, extracellular matrix components and active mediators (such as cytokines, chemokines, growth factors, and humoural factors), in addition to tumour cells. The tumour microenvironment can be divided into an immune microenvironment based on immune cells and a nonimmune microenvironment based on fibroblasts. An abnormal tumour microenvironment is closely related to resistance to cell death, promotion of proliferation, avoidance of immune destruction, maintenance of inflammation or induction of angiogenesis [6]. During autophagy, cytoplasmic material is degraded in lysosomes. Because lysosomes have a distinct membrane as a safety mechanism to prevent the leakage of their degradation enzymes, autophagy involves complex membrane dynamics. There are three types of autophagy involving different modes of delivery of cargo to lysosomes: macroautophagy, microautophagy, and chaperone-mediated autophagy. Macroautophagy is the main regulatory form of autophagy occurring in response to environmental and physiological signals. Microautophagy involves direct phagocytosis of cytoplasmic contents by lysosomes, while chaperone-mediated autophagy involves the translocation of chaperone auxiliary substrate proteins (and potentially DNA and RNA molecules) across the lysosomal membrane [7]. Long noncoding RNAs (lncRNAs) have been widely reported to regulate pathophysiological processes through mechanisms such as gene imprinting, histone modification, chromatin remodelling, transcriptional interference, nuclear transport, transcriptional activation, and cell cycle regulation. LncRNAs are mainly transcribed by RNA polymerase II. LncRNAs are important elements of the mammalian transcriptome that control a variety of cellular mechanisms and regulate cellular processes, such as cell metabolism, drug resistance, growth, proliferation, invasion, metastasis, and apoptosis, ensuring homeostasis. They may be oncogenic or tumour suppressive by directly or indirectly affecting the transcription of a variety of proteins through transcriptional and post-transcriptional changes. The main regulatory mechanisms of lncRNAs are the stabilization of proteins in the nucleus and the sponging of miRNAs in the cytoplasm. They can also act as competitive endogenous RNAs (ceRNAs) by competitively binding to microRNAs (miRNAs) and thus inhibiting their function [8]. In this study, 9 autophagy-related lncRNAs with prognostic value (AC020765.2, AC254562.3, AL031666.1, LINC01426, MMP2-AS1, AC102953.2, AP000695.2, LINC00941 and NKILA) in patients were identified using multivariate Cox regression analysis; a prognostic signature was then established based on these prognostic lncRNAs, which may serve as an independent prognostic factor in lung cancer.

## Materials and methods

### Isolation and sorting of lncRNAs and mRNAs

Data, including transcriptome profile data and clinical information, for all included patients with lung adenocarcinoma and squamous cell carcinoma were downloaded from The Cancer Genome Atlas (TCGA, http://cancergenome.nih.gov/). The data were sorted by a Perl script (https://www.perl.org), and a total of 108 normal samples and 1037 tumour samples were obtained; at the same time, we deleted entries with missing information. Through transferring the annotations to a human genes format, we performed ID conversion and distinguished lncRNAs and mRNAs.

### Autophagy gene and lncRNA screening

The autophagy gene list was obtained from the Human Autophagy Database (HADb, https://autophagy.lu/clustering/index.html). When extracting autophagy genes, we performed an averaging operation on genes that appeared multiple times; normal samples and low-expression genes (autophagy-related mRNAs or lncRNAs with expression < 0.5) were deleted. Pearson correlation analysis was applied to identify correlations between the lncRNAs and autophagy-related genes. A lncRNA with a correlation coefficient |R2| > 0.3 and *P* < 0.001 was considered to be an autophagy-related lncRNA.

### Signature development

Univariate and multivariate Cox regression analysis was performed to evaluate the prognostic value of autophagy-related lncRNAs. To establish the risk score, lncRNAs with a *P*-value < 0.01 in the univariate analysis were included in the multivariate stepwise Cox regression analysis. The following formula was used to determine the risk score for each patient: β gene1 × expr gene 1 + β gene 2 × expr gene 2 + … + β gene n × expr gene n. Cox regression analysis was performed to establish a signature for predicting survival. Specifically, we assigned risk scores by calculating the linear sum of the lncRNA expression levels weighted by the corresponding regression coefficients (β). The β values were calculated by log transformation of the hazard ratio (HR) from the multivariate Cox regression analysis. The high-risk and low-risk groups were established based on the median risk score. The lncRNAs expression values were defined as the expression level of gene n (expr gene n) [9].

### Construction of the lncRNA-mRNA interaction network by cox regression analysis

It was vital to match the autophagy-related lncRNAs and mRNAs according to the Cox regression analysis results; thus, the network visualized with Cytoscape (version 3.7.1) could highlight the connections and mechanisms involved in the development of lung cancer. Furthermore, as the number of lncRNAs was high, it was valuable to create a signature comprising a limited number of variables and the best Akaike information criterion (AIC).

### Gene set enrichment analysis

Gene set enrichment analysis (GSEA) is an method for analysing whole-genome expression profile chip data by comparing genes with predefined gene sets [10]. This method operates through analysis of gene sets and can thus be used to determine whether the gene set shows a statistically significant difference between the two biological states. In this study, we verified whether genes differentially expressed between the two groups are enriched during autophagy.

### Analysis of immune infiltrates

TIMER is a comprehensive resource for systematic analysis of immune infiltrates across diverse cancer types (https://cistrome.shinyappes.io/timer/) and was used to evaluate potential relationships between the risk grouping and tumour-infiltrating immune cells. TIMER employs a recently published statistical method known as deconvolution to deduce the prevalence of TIIGs from gene expression profiles. To approximate the abundance of TIIGs, the TIMER database uses TCGA data for 10,897 samples across 32 types of cancer. To assess the relative variations in gene expression amongst sets in the samples [11, 12], we used a deconvolution algorithm based on gene expression called CIBERSORT (http://cibersort.stanford.edu/). With CIBERSORT, we measured the immune response of 22 TIICs to evaluate their association with the risk grouping in lung cancer and to reveal correlations amongst TIICs. We used standard annotation files to establish gene expression datasets and used the default signature matrix with 1000 permutations. Through Monte Carlo sampling, the approximate *P*-value for the deconvolution was used to determine the confidence levels of the outcomes. To analyse the influence of the high and low risk groupings on the immune microenvironment, we utilized 999 tumour samples that we classified into two groups. To determine the types of lymphocytes affected by the grouping, we set the P-value threshold at < 0.05 [13, 14].

### Statistical analysis

Survival status was the basis for the univariate Cox regression analysis, and R software (version 3.6.2) was used to generate Kaplan-Meier curves. GSEA (http://www.broadinstitute.org/gsea/index.jsp) was used to discriminate two sets of functional annotations. Statistical significance was assumed at a threshold two-tailed *P* < 0.05.

## Results

### Collation of transcriptome data

We identified 14,142 lncRNAs that were extracted from TCGA datasets, and a total of 210 autophagy-related genes were downloaded from the Human Autophagy Database (HADb, http://autophagy.lu/clustering//index.html). We conducted coexpression analysis of the autophagy genes and lncRNAs to identify autophagy-related lncRNAs (|R2| > 0.3 and *P* < 0.001). According to the properties of the genes, we separated 1496 identified lncRNAs for further identification of prognostic genes. We combined the two data types futime (survival time) and fustate (survival status), which were obtained from the clinical data (downloaded from TCGA), into the lncRNA expression matrix. Patients with incomplete clinical information (futime, fustate, age, sex, grade, state or TNM) were excluded from the following analysis.

### Construction of the cox prognostic model

**Fig. 1:**
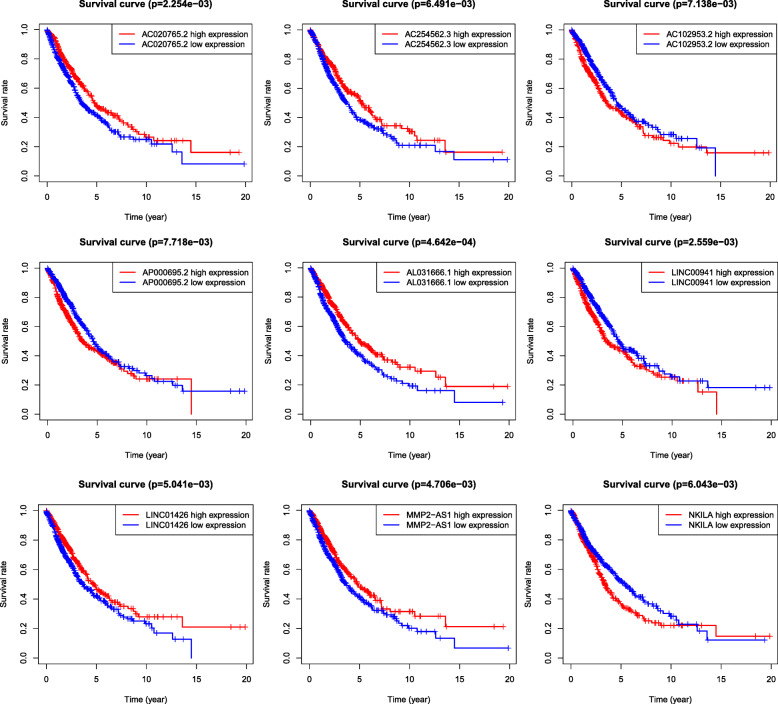
Construction of prognostic signature. The red curve in the survival curve represented the high expression of lncRNA, and the blue curve represented the ground expression of lncRNA. The low expression of five lncRNAs related to autophagy had better survival, while the high expression of the other four lncRNAs related to autophagy had better survival

Through univariate Cox regression analysis, 18 lncRNAs were found to have prognostic value for lung cancer (*P* ≤ 0.01), and these lncRNAs were subjected to multivariate Cox regression analysis. A risk score formula based on AC020765.2, AC254562.3, AL031666.1, LINC01426, MMP2-AS1, AC102953.2, AP000695.2, LINC00941 and NKILA had the lowest AIC (Akaike information criterion); among these lncRNAs, five were favourable factors (AC020765.2, AC254562.3, AL031666.1, LINC01426, MMP2-AS1) and four were considered unfavourable prognostic factors (AC102953.2, AP000695.2, LINC00941 and NKILA). The risk assessment score for the prediction of overall survival was calculated as follows: expAL031666.1 × 0.176009 + expAC020765.2 × 0.138779 + expAC102953.2 × 0.103983 + expAP000695.2 × 0.145198 + expNKILA × 0.048298 + expMMP2-AS1 × 0.187273 + expLINC01426 × 0.086274 + expAC254562.3 × 0.150477 + expLINC00941 × 0.054391 (Fig. 1) [15, 16].

### Visualization of co-expression network

**Fig. 2:**
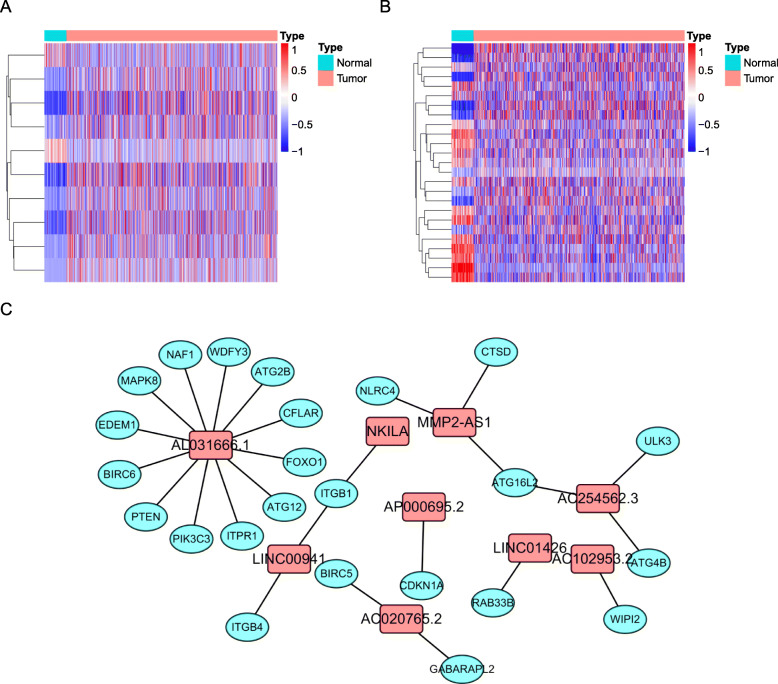
Co-expression network construction. (**A**) Expression of lncRNA related to autophagy in the prognostic signature of heat map between normal and tumor samples. (**B**) Expression of mRNA related to autophagy in the co-expression network in heat map between normal and tumor samples. Red represented high expression, blue represented ground expression, and the depth of color represented the level of expression. (**C**) The co-expression network showed the link between lncRNA and autophagy-related mRNA

**Fig. 3:**
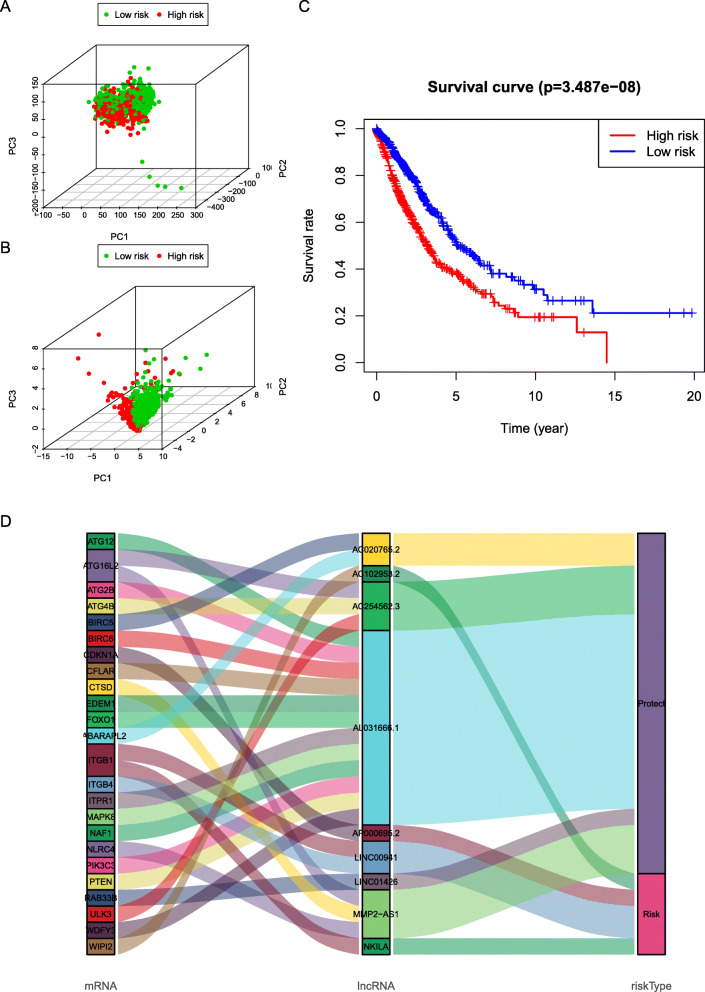
Presentation mode of the prognostic signature. (**A**) Principal component analysis diagram (PCA) showed the distribution of all genes. (**B**) Principal component analysis chart (PCA) showed the distribution of prognostic signature. Green represented low risk, and red represented high risk. (**C**) Kaplan-meier curve reflected the survival significance of the prognostic model. (**D**) The alluvial map was used to further show the relationship between autophagy-related mRNA and autophagy-related lncRNA, and their risk types

To better present the connections among and mechanisms linking prognosis-related autophagy lncRNAs and mRNAs, we first visualized the coexpression results with Cytoscape and constructed heat maps for the lncRNAs and mRNAs in the coexpression network to show the differences in the expression data (Fig. 2A-C). The distribution of the different patients, who were separated into two groups by the median value of the risk score, was significantly different in Cox regression analysis of the autophagy-related genes, while it was not significantly different between the two groups in the Cox regression analysis of all genes (Fig. 3A-B) [17]. The risk survival curves indicated that the five-year survival rates in the low-risk (CI: 0.446–0.579) and high-risk (CI: 0.32–0.443) groups were higher than 0.5 and 0.38, respectively (*P* < 0.0001) (Fig. 3C). Next, we constructed a Sankey diagram to further classify the lncRNAs as protective lncRNAs (the higher the expression of the lncRNA, the lower the risk) or risky lncRNAs and more comprehensively visualize their connections (Fig. 3D).

### Validation of the cox prognostic model

**Fig. 4:**
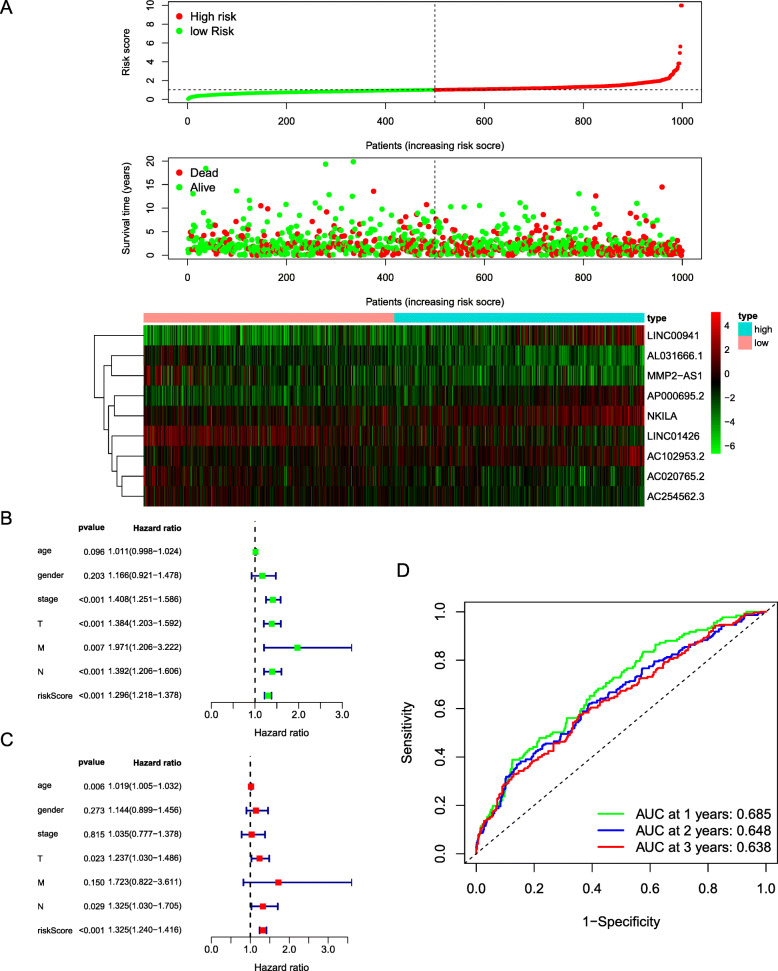
Validation of prognostic signature. (**A**) The risk plot showed that as the risk score increases, the proportion of patient deaths increases, which in turn indicateed the reliability of the model. The risk heat map further showed the difference in the expression of prognostic lncRNA between the high and low risk groups. (**B**) Univariate Cox regression analysis showed that stage, T, N, M and riskscore had prognostic significance for non-small cell lung cancer. (**C**) Multivariate Cox regression analysis displayed that age, T, N and riskscore were independent prognostic factors

**Fig. 5:**
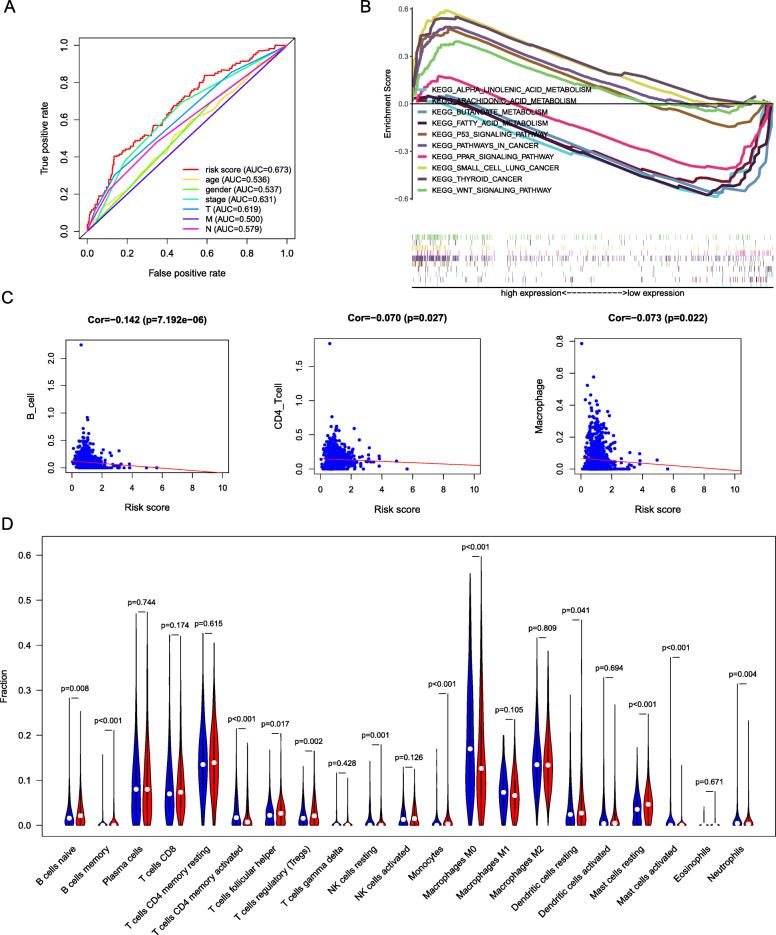
Immune Infiltration Analysis. (**A**) The area under the ROC curve was used to predict the predictive power of the prognostic model. The largest area under the prognostic model curve indicated the best prediction effect. (**B**) KEGG enrichment analysis showed that the prognostic model is mainly enriched in the cancer pathway. (**C**) The scatter plot showed that the prognostic model is significantly correlated with B cells, CD4+ T cells and macrophages. (**D**) The violin chart was used to show the difference between the 21 groups of immune cells in the high and low risk groups. Red represents high expression and blue represents low expression

The risk curve had three parts, and the patients’ risk increased sequentially from left to right. The first part implied that we divided these patients into the high-risk and low-risk groups on the basis of the median value of the risk score. The survival status plot indicated that as the risk value increased, the patients’ survival time decreased. The expression heat map indicated that risk increased with increased expression of some lncRNAs (LINC00941, AP000695.2, NKILA and AC102953.2) and with decreased expression of other lncRNAs (AC020765.2, AC254562.3, AL031666.1, LINC01426 and MMP2-AS1) (Fig. 4A). To evaluate whether the constructed model was independent of other clinical traits as a predictive factor, we performed an independent prognostic analysis. It was found that clinical stage (*P* < 0.001), T stage (*P* < 0.001), M stage (*P* = 0.007), N stage (*P* < 0.001) and risk score (P < 0.001) were directly related to the prognosis of patients (Fig. 4B), while multivariate Cox regression analysis showed that only T stage (*P* = 0.023), N stage (*P* = 0.029) and risk score (*P* < 0.001) were statistically independent predictive factors (Fig. 4C) [18]. The prediction efficiency of the model was evaluated by ROC curve analysis, and it can be seen that the area under the curve was 0.685 for one-year survival, 0.648 for two-year survival, and 0.638 for three-year survival. This finding indicates that the predictive efficiency of our model is good (Fig. 4D). The area under the red curve in the ROC curve with multiple indicators was 0.673, suggesting that our model had promising power for predicting the clinical outcome of patients. Furthermore, among the clinical traits, risk score had the largest area under the curve value; thus, our model was also superior to other clinical traits for predicting the survival of patients (Fig. 5A) [19].

### Gene set enrichment analysis

Further functional annotation was conducted through GSEA, and the results revealed that the differentially expressed genes between the two groups were enriched in metabolism-related and tumour-related pathways. GSEA showed that a total of 45 gene sets were significantly enriched with a nominal *P* value < 0.05. Among the identified pathways, several pathways were well established in cancers, including small cell lung cancer, pathways in cancer, thyroid cancer, P53 signaling pathway, and WNT signaling pathway; all of these pathways promote the development of tumours, indicating that these pathways were active in high-risk patients, while other pathways were silent in high-risk patients. The PAR signalling pathway participates in lipid metabolism and induces anticancer effects in human tumours. Butanoate metabolism, which significantly increases the metabolic stress on tumour cell mitochondria, promotes specific apoptosis of lung cancer cells and inhibits tumour growth. Fatty acid metabolism, α-linolenic acid metabolism and arachidonic acid metabolism inhibit the initiation and metastasis of cancer (Fig. 5B) [20, 21].

### Differences in immune cells in the high- and low-risk groups

**Fig. 6:**
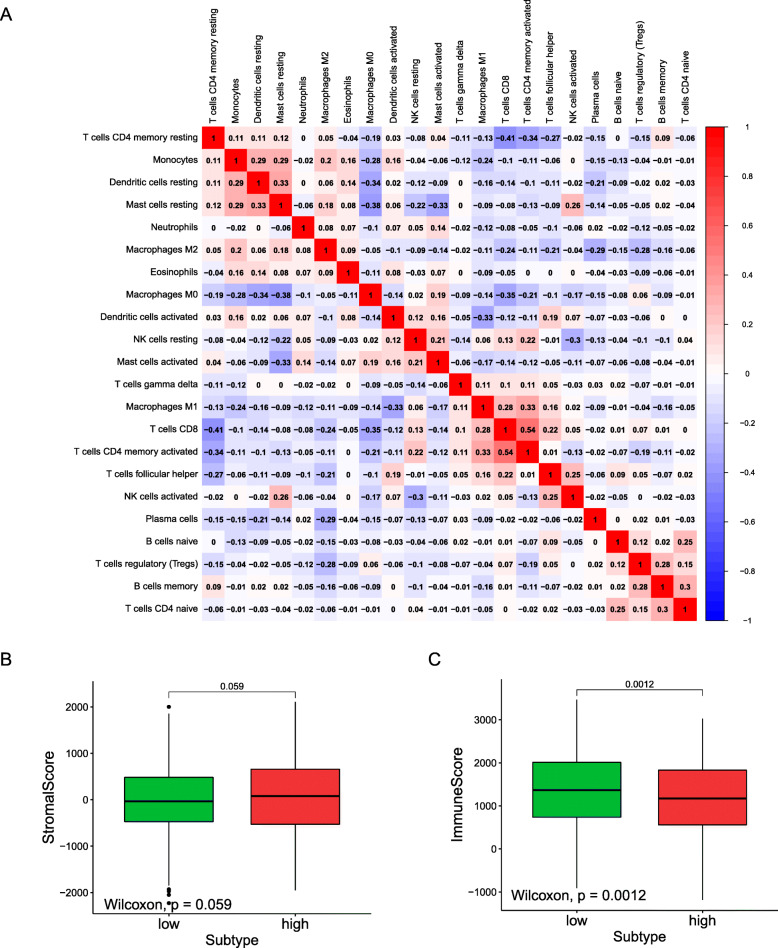
Immune microenvironment analysis. (**A**) Correlation heat map showed the correlation between 22 immune cells and prognostic models. (**B**) We predicted the content of stromal cells through matrix scores, and then analyzed the differences in stromal cells between high and low risk groups. (**C**) We used the immune score to predict the content of immune cells, and then analyzed the differences in immune cells in the high and low risk groups

Independent tumour-infiltrating lymphocytes contribute to the prediction of overall survival and the status of sentinel lymph nodes. Hence, TIMER was applied to analyse the possible relationships between risk grouping and immune infiltration in lung cancer. As shown in Fig. 5C, a negative correlation existed between risk classification and the numbers of B cells (*P*-value = 7.192 × 10–6), macrophages (*P*-value = 0.022) and CD4+ T cells (*P*-value = 0.027) [22, 23]. To estimate whether there is a difference between the tumour immune microenvironment in the two groups of patients, 999 tumour patients were divided into a low-risk group and a high-risk group, which contained 500 and 499 patients, respectively. The comprehensive CIBERSORT algorithm was employed to characterize the infiltration of 21 different immune cells based on gene expression profiles, with patients separated into two groups based on the median risk score. Markers of activated memory CD4+ T cells and M0 macrophages showed low expression in the low-risk group, while markers of resting mast cells showed high expression in the low-risk group (Fig. 5D). The correlation heat map obtained with the 22 types of immune cells revealed that CD8+ T cells correlated positively with activated memory CD4+ T cells but showed a negative relationship with resting memory CD4+ T cells (Fig. 6A). The immune score and matrix score of the patients can help to determine the degree of immune cell infiltration in the tumour microenvironment and the tumour purity. Analysis of the matrix microenvironment showed that there was no significant difference among the patients when they were divided into two groups based on the median value of the matrix score, while when the patients were divided into two groups based on the median value of the immune score, significant differences were revealed in the immune microenvironment (Fig. 6B-C) [24–27].

### Correlation analysis of clinical parameters

**Fig. 7:**
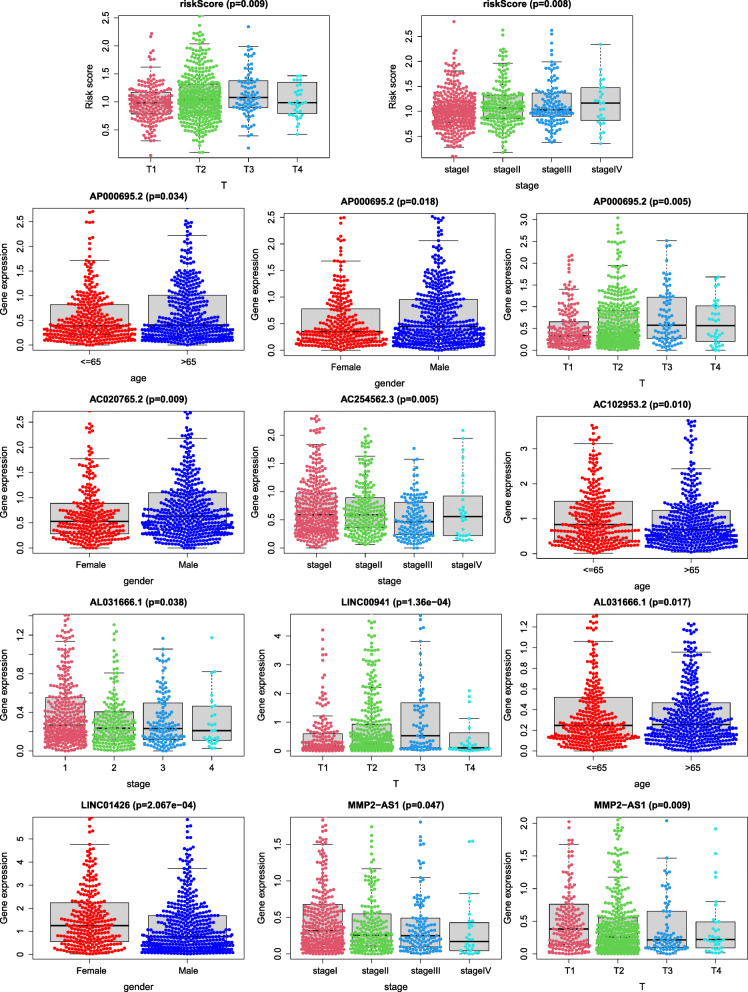
Clinical correlation analysis. The prognostic model was significantly correlated with T and stage, which implied the accuracy and reliability of the model. While Part of the lncRNAs in the model were also significantly related to clinical factors respectively

We conducted correlation analysis of clinical parameters to assess whether the risk score and the lncRNAs in the Cox prognostic model are related to clinical traits. We found that the risk model was significantly related to clinical stage and T stage. In addition, AP00695.2 in the model was significantly related to age, sex and T stage, while AC020765.2 was related to sex, AC254562.3 was related to clinical stage, AC102953.2 was significantly different between ages, and LINC00941 was related to T stage. The differences were significant: AL031666.1 was significantly different between ages and stages, LINC01426 was significantly different between sexes, and MMP2-AS1 had an obvious difference between clinical stages and T stages (Fig. 7).

## Discussion

Lung cancer, which is one of the most fatal malignancies, is currently a major health issue worldwide. It is of great urgency to find a way to predict the overall survival rate of patients with lung cancer. Epigenetic modifications of genes, especially lncRNAs, have shown close links to lung cancer [28, 29]. LncRNAs, as supplemental genes/miRNAs, are promising predictors of the risk of lung cancer recurrence. Due to technological limitations, problems still exist in functional research lncRNAs in comparison with coding RNAs. Therefore, it is vital to establish a risk model to better predict the prognosis of lung cancer.

In this research, we identified 9 prognostic autophagy-related lncRNAs and divided patients into high-risk and low-risk groups based on the median risk score. Through univariate and multivariate Cox regression analysis, we concluded that the risk model is an independent prognostic factor.

Autophagy originates with the formation of membrane structures called phagocytic cells or membranes. After phagocytic cell formation begins, the double membrane grows to surround the cellular contents during a process called the autophagic extension phase. Autophagy can promote the survival of tumour cells but can also lead to cell death. It can be enhanced or inhibited by anticancer agents. Upregulation of autophagy during cancer treatment can promote either the survival or death of tumour cells. Although little is known about the role of autophagy in cancer therapy to date, recent studies have suggested that therapeutic autophagy will become a new approach for lung cancer treatment [30–32]. First, autophagy can have a tumour-suppressive function. Autophagy is a valuable mechanism used by cells to maintain cell integrity and genome stability. The absence of autophagy genes naturally interferes with this homeostasis; thus, it may initiate cell tumour development. Furthermore, a variety of autophagy mechanisms contribute to tumour suppression. Under stress, autophagy is activated to remove damaged proteins and organelles, including mitochondria. Inhibition or lack of autophagy leads to an increase in the reactive oxygen species level, resulting in accumulation of DNA damage, which is manifested as gene amplification, increased numbers of double-strand breaks and polyploid nuclei. This increase in DNA damage may lead to higher susceptibility to the onset and development of cancer.

In addition, autophagy is a carcinogenic process, and both mechanical tissue and genetic research support this hypothesis. When the intracellular and extracellular environments are deficient and cells are under metabolic stress, autophagy is activated as an adaptation mechanism. In the early stages of tumour formation, cancer cells often experience hypoxia and an environment in which nutrients are limited due to tumour growth because of the lack of an effective blood supply. These conditions cause metabolic stress and lead to reduced mitochondrial oxidative phosphorylation. Subsequently, cancer cell proliferation is suppressed, and cells can enter a dormant state. During dormancy, tumour cells rely on autophagy as a survival strategy, thereby repurposing nutrients to promote cell survival. When the environmental stress is ameliorated, cancer cells can resume proliferation. In fact, defective autophagy causes lung tumours to halt progression and become benign eosinophilic tumours, which are characterized by an abundant cytoplasm and high mitochondrial quality [33]. Recent studies have shown that LINC01426 can act as a predictive gene of SQCLC and GC. LINC00941 was defined as an optimal diagnostic lncRNA biomarker for HNSCC, GC and LUAD. AP000695.2 was used as one of the indicators for constructing a prognostic model of gastric adenocarcinoma [34]. NKILA was found to promote tumour immune evasion by sensitizing T cells to activation-induced cell death [35]. NKILA was also found to suppress nasopharyngeal carcinoma carcinogenesis and metastasis via NF-kappaB pathway inhibition [36]. NKILA was shown to suppress TGF-beta-induced epithelial-mesenchymal transition by blocking NF-kappaB signalling in breast cancer [37].

Equally important, our study used the TIMER database to reveal connections between the risk signature and immune infiltration levels in lung cancer. We found that the associations of the risk signature with B cells, CD4+ T cells and macrophages were the strongest. Moreover, our CIBERSORT analysis revealed that the expression of markers of activated mast cells, M0 macrophages and activated memory CD4+ T cells was increased in the high-risk group, whereas the expression of markers of naive B cells, T follicular helper cells, resting dendritic cells and resting mast cells was decreased. Our results could indicate a possible mechanism by which the lncRNAs in the risk signature regulate the functions of mast cells in tumours. Mast cells are multifunctional cells, and related studies have confirmed that they are related to the pathological process of neoplastic diseases. For example, mast cells can promote tumour angiogenesis by releasing heparin or dissolve surrounding connective tissue by releasing proteolytic enzymes, which is beneficial for tumour growth and metastasis. In contrast, other studies have shown that mast cells surrounding the tumour have the role of tumour defence and host protection. Combining previous findings with our research, we can propose a corresponding explanation that activated mast cells promote tumour growth and resting mast cells inhibit tumour growth.

In conclusion, we demonstrated that the lncRNAs investigated using this model can serve as therapeutic targets for precision treatment of lung cancer. Practical research will be conducted to further verify their biological functions and explore the underlying molecular mechanisms.

## Data Availability

All datas were from TCGA databases. Which are publicly available (https://www.cancer.gov/about-nci/organization/ccg/research/structural-genomics/tcga).
